# Fitting a square peg in a round hole? A mixed-methods study on research ethics and collaborative health and social care research involving ‘vulnerable’ groups

**DOI:** 10.1186/s12961-025-01290-3

**Published:** 2025-04-01

**Authors:** Chiara De Poli, Jan Oyebode, Mara Airoldi, Martin Stevens, Andrea Capstick, Nicholas Mays, Michael Clark, Annelieke Driessen, Carol Rivas, Bridget Penhale, James R. Fletcher, Amy M. Russell

**Affiliations:** 1 Care Policy and Evaluation Centre, London School of Economics and Political Science, Houghton Street, London, WC2A 2AE United Kingdom; 2 Centre for Applied Dementia Studies, Faculty of Health Studies, University of Bradford, Richmond Road, Bradford, BD7 1DP United Kingdom; 3 Blavatnik School of Government, Radcliffe Observatory Quarter, University of Oxford, 120 Walton St, Oxford, OX2 6GG United Kingdom; 4 The NIHR Policy Research Unit in Health & Social Care Workforce, King’s College London, Virginia Woolf Building, Kingsway, London, WC2B 6LE United Kingdom; 5 Department of Health Services Research and Policy, London School of Hygiene & Tropical Medicine, 15-17 Tavistock Place, London, WC1H 9SH United Kingdom; 6 Anthropology Department, University of Amsterdam, Nieuwe Achtergracht 166, 1018 WV, Amsterdam, The Netherlands; 7 Social Research Institute, University College London, 27 Woburn Square, London, WC1H 0AA United Kingdom; 8 School of Health Sciences, University of East Anglia, Norwich, NR4 7TJ United Kingdom; 9 School of Management, University of Bath, Convocation Avenue, Claverton Down, Bath, BA2 7AY United Kingdom; 10 Faculty of Medicine & Health, Leeds Institute of Health Sciences, University of Leeds, Leeds, LS2 9NL United Kingdom

**Keywords:** Research ethics, Collaborative research, Participatory research, Co-creation, Co-design, Co-production, ‘Vulnerable’ groups, Research ethics committee, Institutional review board, Procedural ethics

## Abstract

**Background:**

Current research ethics frameworks that oversee health and social care research, in the United Kingdom and internationally, originated in biomedical research, having positivist underpinnings and an orientation towards experimental research. Limitations of these frameworks have been extensively documented including with regard to health and social care research that adopts collaborative approaches. This article contributes to debates about how the research ethics system deals with collaborative research with groups labelled or potentially perceived as vulnerable, and identifies practical recommendations to ensure a better fit between principles and practices of research ethics and those of collaborative research.

**Methods:**

We conducted a two-round online Delphi study with 35 academic researchers with experience of collaborative research involving vulnerable groups and of seeking research ethics approval in England (United Kingdom), followed by a focus group with eight members of the Delphi panel. The Delphi questionnaire, organised in 12 themes, comprised 66 statements about how researchers experience research ethics review and how the research ethics system could be improved. The focus group discussed the results of the Delphi study to generate practical recommendations.

**Results:**

By the end of the second Delphi round, only one statement relating to the experience of the current research ethics system reached consensus, signalling heterogeneous experiences among researchers working in this field. A total of 32 statements on potential improvements reached consensus. The focus group discussed the 14 Delphi statements with the highest levels of consensus and generated 12 practical recommendations that we grouped into three clusters (1. Endorsing the ‘collaborative’ dimension of collaborative research; 2. Allowing flexibility; and 3. Strengthening the relational and ongoing nature of ethical research practice).

**Conclusions:**

This work provides further empirical evidence of how the research ethics system deals with collaborative research involving ‘vulnerable’ groups. It also offers practical recommendations to ensure that the collaborative dimension of such research receives proper ethical scrutiny, to introduce a degree of flexibility in research ethics processes and supporting documents, and to replace formal, one-off research ethics approvals with ongoing, situated, relational ethical processes and practices.

**Supplementary Information:**

The online version contains supplementary material available at 10.1186/s12961-025-01290-3.

## Introduction

Research ethics and governance provides the regulatory and institutional cornerstone for the conduct of contemporary research involving human participants, in the United Kingdom and internationally. Although current research ethics frameworks were originally developed to regulate biomedical, experimental research, over time their application has been expanded to also regulate social sciences.

Such expansion of research ethics oversight beyond its original scope has reportedly shown a range of limitations and caused a number of negative or unwanted consequences, in particular in the case of research using non-experimental, naturalistic designs. Health and social care research that adopts participatory and collaborative approaches and involves participants who are commonly positioned as ‘vulnerable’ within contemporary discourses in research ethics and governance systems is a case in point and is the focus of our work.

The study that we have conducted and that we report here aims to progress the debates about the limitations of the current research ethics system and its unintended consequences on participatory and collaborative research with ‘vulnerable’ groups and to make practical suggestions about how implicated systems could be improved. It does so by exploring the topic from the perspective of various stakeholders of the research ethics system. Here we report on the first part of the study, involving academics and researchers. The second part will include research participants from perceived vulnerable groups, research funders and research ethics committees and will be reported in a future companion article.

For the purposes of this work, participatory and collaborative research (‘collaborative’ from here on) is defined as research in which participants are actively involved in shaping the research, beyond simply providing data. This includes participatory action research, community-based participatory research and collaborative designs (e.g. co-creation, co-design, co-production). Regardless of the specificities of each term, the umbrella of collaborative research moves away from the traditional divide between knowledge producers (typically academic researchers) and knowledge users (such as policy-makers, service providers or the public) and from the primacy of academic knowledge against other types of knowledge (e.g. tacit knowledge or lived experience). For these reasons it has been promoted and used to seek to increase research relevance, close the evidence-to-practice gap and promote inclusion in research, creating a stage for ‘under-served’ and ‘seldom-heard’ groups to voice what matters to them [1].

The concept of ‘vulnerability’ is not generally well defined by research ethics policies and guidelines, but it is operationalized as a category for risk assessment in ethical review [2, 3]. In practice, individuals presenting with a condition, disease or disability are assumed to be vulnerable and in need of additional protection during research participation. This blanket, unproblematized conceptualization of vulnerability may limit opportunities for voices to be heard in research, which not only exacerbates social exclusion, but also affects the trustworthiness of the resulting research.

This work assumes that vulnerability may be perceived rather than real, is individual and situational, and is to be evaluated with respect to the context and intersectional identities of individual research participants. Hence, individuals may be rendered vulnerable by personal, social, political and environmental conditions interacting with disease, disability or other factors, which together may affect their decision-making or place them at higher risk of harm, undue influence, coercion or exploitation. Our choice of using the term vulnerability is in keeping with the language currently used in research ethics policies and regulations [2]. We acknowledge that this choice can be disputed and viewed as contentious. It may be seen as perpetuating the power and knowledge dynamics that collaborative research aims to readdress. Nevertheless, we argue that this work can contribute to the ongoing debate around reframing vulnerability in the context of research and to raising awareness around language and meaning.

## Background

Research is characterized by asymmetrical power relationships between researchers and research participants and by a tension between the risks that participants may incur vis-à-vis the potential benefits of research for individuals or society at large. Research ethics frameworks grounded in the principle of respect for autonomy, justice, beneficence (i.e. to do ‘good’) and non-maleficence (i.e. to do no harm) have been developed to support ethical oversight by research ethics bodies [i.e. Research Ethics Committees (RECs) in the United Kingdom, Institutional Review Boards (IRBs) in the United States and Canada], and safeguard research participants whilst offering ethical guidance for researchers [4, 5]. Guidelines such as the Nuremberg code (1947) [6], the Declaration of Helsinki (1964) [7] and the Belmont Report (1978) [8] represented the first attempts of the international community to tackle research scandals and ethical violations that took place from the 1930s through to the 1970s. Over time, this body of international regulation and guidance has not only been revised and updated [9], but also progressively expanded (e.g. the International Ethical Guidelines for Biomedical Research Involving Human Subjects of the Council for International Organizations of Medical Sciences [10]) and complemented by additional national or regional regulation (e.g. the EU directive on medical trials) [2].

Whilst the approach to research ethics has remained grounded in the core set of standards and obligations originally developed in the context of biomedical, experimental research (e.g. fair participant selection, favourable risk–benefit ratio, independent review, informed consent), its application has progressively expanded to non-biomedical, social sciences research, using non-experimental research methods [11] - a process described as ‘ethics creep’ [12].

Although this expansion aimed to guarantee adequate protection for participants, regardless of the type of research, the application of these standards beyond the original biomedical field has proved problematic.

On a theoretical level, it has revealed profound methodological and paradigmatic clashes between what might be generalized as positivist and non-positivist research [13, 14]. More specifically, these tensions often emerge from different design types, particularly experimental designs (which are minutely pre-determined) and naturalistic designs (which are more unpredictable). These design types mostly align with biomedical positivist research (experimental) and social scientific non-positivist (naturalistic) forms of research, but we must acknowledge that this alignment is not absolute.

This fundamental tension between experimental predictability and naturalistic unpredictability has been intensified by higher education’s financialization during the 21st century. As with many large businesses, administrative systems in universities and health research institutions prioritize risk elimination by maximizing predictability. This has led to the contemporary emphasis on risk management and a legalistic approach to research ethics [15, 16]. Hence, the traditional critique of research ethics as being too biomedical is perhaps better understood as a problem emerging from naturalistic empiricism being fundamentally at odds with risk-averse financialized institutions. The result is typically a poor fit between research ethics arrangements and social sciences research, which has been repeatedly critiqued [17].

Research that adopts participatory and collaborative approaches, often using qualitative or mixed-methods [18], which has become increasingly common in health and social care research, has been particularly disadvantaged by the dominant, biomedicalized and corporatized research ethics frameworks [19]. Although the fundamental principles of research ethics are universally shared across disciplines, epistemological stances and research methodologies, six different tensions are apparent in how researchers, research ethics institutions and research participants interpret and operationalize these principles in the context of collaborative research.

First, the characteristic emergent nature and iterative design of collaborative research clashes with research ethics institutions’ requirements that expect research to be outlined ex ante and in full in detailed protocols [20] to ensure fidelity of research activities and to avoid any prospective reputational and/or financial risk to research institutions [21].

Second, the medical gaze of much research ethics shapes the appraisal of risks and benefits to the participants contributing to participatory and collaborative research [19]. Risks to the participants may be over-emphasized whilst potential benefits for them may be discounted, and full consideration is not given to the potentially complex emotional reactions of the researcher(s) (e.g. if exposed to emotionally charged, distressing or compromising participants’ narratives) [22, 23].

Third, collaborative research may raise issues related to privacy, confidentiality and anonymity, which attract different interpretations from researchers, participants and research ethics institutions [24, 25]. Research ethics bodies often place great emphasis on a blanket approach to protecting individual identities, an approach often opposed by research participants who perceive it as unnecessary, paternalistic or disempowering, and who would prefer recognition for their contributions to research [26, 27]. Furthermore, the requirement to state intentions for anonymity at the design and approval stage means that participants do not have time to understand the implications of what waiving anonymity might mean, issues that may be better negotiated in the full course of a collaborative research project.

Fourth, and linked to the above, collaborative research is highly relational, often creating tight ties between researchers and research participants, potentially making formal advance consent-seeking unworkable, and forging relationships that blur the boundaries of professional and personal relationships [12, 25, 28]. In addition, consent is framed as a one-off, static concept, rather than as an ongoing, relational, and situational process [29–31].

Fifth, collaborative research is often utilized to open up the research process to under-represented and seldom-heard groups, often labelled or perceived as vulnerable. However, the conceptualization of vulnerability, as noted, is far from clear. It spans a continuum from identifying all research participants as vulnerable (an implicit assumption made by the Nuremberg code) to specifically labelling particular people or groups whose characteristics may make them susceptible to specific kinds of harm or exploitation. The latter is the explicit view taken in the Belmont report, where racialization, economic disadvantage, serious sickness and institutionalization are identified as specific markers of vulnerability [32]. Research ethics policies rarely provide an explicit definition of vulnerability (unsurprisingly given its normativity and context-dependence) and, more often, rely on implicit assumptions and on labelling vulnerable groups on the basis of sources of vulnerability [2]. Again, vulnerability is characterized as static and categorical, rather than an evolving, relational phenomenon.

Lastly, the increasing use of rapid and online research methods, whilst providing a practical strategy to carry out research in a fast-paced and changing environment or to support policy and decision-making in real time, has further exacerbated all these issues. It is difficult to reconcile rapid research with the often lengthy bureaucratic ethical approval procedures through which ethical governance is performed [33–35].

Research at the intersections of these dimensions, using emerging, collaborative research designs and involving groups labelled as vulnerable (e.g. adults who lack or have fluctuating capacity to consent [36, 37], individuals receiving palliative care [38], frail and older people [39], children [40, 41], young people with adverse childhood experiences [42], people with disabilities [43], adoptees [15], prisoners [44]) is cumulatively affected by these issues.

Such issues have been documented mostly from the perspective of researchers who feel that the system prevents a thoughtful and ongoing engagement with the ethical issues surrounding the specific research studies [24, 45, 46] and have described their experience of navigating the research ethics system as ‘jumping through hoops’, ‘walking a tightrope’ or ‘something to get through’ [24]. Likewise, REC members, albeit more rarely, have also expressed dissatisfaction with the institutional and practical constraints within which they are expected to operate when reviewing collaborative research, and have described their experiences of navigating the review process from their perspective [47, 48].

Participants’ experience of research and understanding of research ethics in the context of collaborative research specifically, and qualitative research more broadly, has been sparsely investigated [49–54]. The common perception is that RECs, at times, have a paternalistic attitude towards research participants, with the risk of depriving them of individual agency and restricting rather than enabling their opportunity for participation [49, 55].

Alternative, informal research ethics frameworks, developed with a bottom-up approach by individuals with experience of being research participants, have started to emerge in response to the issues of the traditional research ethics framework [56, 57]. Such frameworks, which can be adopted on a voluntary basis, are not formally recognized as a viable review process for institutionally aligned researchers, with the consequence that their actual impact is hindered.

Academic researchers have also made some sparse attempts to start reconciling the paradigmatic clash between collaborative research and research ethics to move away from a transactional, regulatory, procedural understanding of ethics towards a relational, context-sensitive approach, and to complement an ethics of principle with an ethics of care. Good practices or heuristic models that RECs/IRBs and researchers may wish to consider are now available (e.g. [43, 58, 59]). Nevertheless, issues are still apparent: approval of collaborative research is often delayed [48], recruitment of research participants from ‘vulnerable’ groups is hindered [60], more conservative, traditional designs and methods are preferred over methodological innovations [11] and ‘ethics-in-practice’, the actual ethical conduct of the research project, can be overlooked [61].

In view of this context, we have undertaken a mixed-methods study to generate consensus on changes to the research ethics system that could ensure a better fit between the normative principles underpinning policy and regulatory frameworks of research ethics and the related operational processes and the aim and methodologies of collaborative research involving ‘vulnerable’ adult populations.

The study was organized in five consecutive phases. Phase 1 (analysis of UK policy documents and guidelines about the research ethics framework), phase 2 (scoping review of relevant literature), and phase 3 (exploratory focus group with active academic researchers with experience of working in this field in England, United Kingdom) have been reported elsewhere [62] and were preparatory to the Delphi study that followed (phase 4). The two-round, online Delphi study involved academic researchers with experience in conducting collaborative research with vulnerable groups in England (United Kingdom) and aimed to generate consensus on what changes should be considered to ensure a better fit between the principles and processes of the research ethics system and the aims and practices of collaborative research. A final focus group with academic researchers identified from the Delphi panel was conducted to inform practical recommendations and assess their expected impact (phase 5). This article reports the last two phases of the study.

## Methods

### Delphi study

The Delphi technique is a well-established approach to elicit expert judgements and transform individual opinions into a group consensus [63]. It uses a structured process by which a series of questionnaires (‘rounds’) is administered to gather information from participants until ‘group’ consensus is reached [64]. Aggregated group answers from the previous round are supplied to the participants in each subsequent round. This gives participants the opportunity to revise or confirm their judgments on the basis of the anonymized opinions of others from the previous round [65]. Answers which reach a pre-determined consensus threshold are excluded from subsequent iterations.

The Delphi study was conducted with a purposeful sample of academic researchers with experience and expertise of carrying out collaborative research with ‘vulnerable’ populations in England (United Kingdom).

A total of 52 participants from 26 English universities were identified or snowballed from the research team’s professional networks and from participants in the exploratory focus group (phase 3). Potential participants were invited by email. To help them determine whether they had relevant experience and expertise to take part in the survey, and to ensure a shared understanding of the scope of the work, we provided working definitions of key terms within the survey questionnaire, as follows:

- *Vulnerable population*. A clear and definitive definition of what a vulnerable population is or how it can be identified is lacking. In line with previous work, in this study we take the view that vulnerability is individual and situational, and as such, should be evaluated with respect to the context and to the characteristics of individual research participants. Following on from this, vulnerability is not a quality of an individual, rather, individuals may be rendered vulnerable by disease or disability or by personal, societal or environmental conditions which may affect their decision-making or may make them at higher risk to harm or to undue influence, coercion or exploitation.
- *Collaborative research.* We refer to collaborative research in a broad sense to include participatory action research, community-based participatory research and collaborative research approaches such as co-production, co-design and co-creation, in which participants are involved in an active way, beyond simply providing data.

The work conducted in phases 1–3 of the study [62] informed the development of the survey statements, which were organized into 12 thematic sections. The analysis of UK research ethics policies and documents available from the Health Research Authority (HRA) (phase 1) had identified 12 themes, along with the associated principles and the processes that operationalize these principles. The results of this work were used to develop a set of statements with the stem ‘The current research ethics system…’ to describe how the system currently works or is expected to work. 

A second set of statements with the stem ‘The research ethics system needs to…’ described options for improving how the research ethics system deals with collaborative research with vulnerable groups. These had been identified in the scoping review of the literature and the subsequent focus group, conducted during phases 2 and 3 of the study, respectively [62].

A draft of the questionnaire was developed by C.D.P. and reviewed with J.O. to ensure that each statement was categorized under the most suitable theme, achieving consistency and avoiding duplication. Instances of uncertainty or ambiguity were addressed and resolved through discussion. As a result of this process, we generated 27 statements with the first stem and 39 statements with the second stem, all of which were included in the questionnaire. Please see Additional files for the questionnaire.

The Delphi study took place online in two rounds (July and December 2020) and was delivered using Qualtrics (https://www.qualtrics.com). At each round, participants were asked to complete an online questionnaire within four weeks of receipt. Participants were invited to express their level of agreement with each statement using a 5-point Likert scale (strongly disagree, disagree, neither agree nor disagree, agree, strongly agree). The questionnaire was supplemented by explanatory notes to set out the background for each theme and provide the evidence base (derived from phases 1–3 of the study) underpinning each statement. We considered consensus to be reached if 80% of respondents expressed strong agreement/agreement or strong disagreement/disagreement for a statement.

### Final focus group

All participants in the second round of the Delphi study were invited to participate in a final focus group. Among them, eight participants confirmed their availability for and attended the 2-h online focus group that we held in March 2021 (Table 1).

**Table 1:** Participants in the final focus group

Participant ID	Gender	Years of experience	Research interest
FG2-1	Female	5–10 years	Dementia care
FG2-2	Male	More than 10 years	Health services
FG2-3	Female	5–10 years	Palliative care
FG2-4	Male	More than 10 years	Health and social care ethics
FG2-5	Female	0–5 years	People with intellectual disabilities
FG2-6	Male	0–5 years	Dementia care
FG2-7	Female	More than 10 years	People with hidden disabilities
FG2-8	Female	More than 10 years	Older people

Using the sub-set of statements which gained the highest consensus in the Delphi study (i.e. above 90% consensus on the same level of agreement), the focus group aimed to (1) generate practical recommendations that could help ensure that the research ethics system is better fit for purpose when dealing with collaborative research with groups deemed ‘vulnerable’ and (2) prioritize such recommendations on the basis of their expected impact on collaborative research involving such groups.

The facilitation was supported by an online tool that allowed recording of the group’s recommendations and, for each, the expected impact was recorded using a slider bar with anchor points 0, 50 and 100. The group worked through the full list of statements, starting from the one with the highest consensus, and identified recommendations that could impact practice. When the discussion generated more than one recommendation for a statement, all were noted and scored using the online tool. As the discussion progressed, some recommendations were reworded to improve clarity or merged to avoid duplications. Participants were invited individually to score each recommendation then agree collectively on a final score. By the end of the discussion, 12 recommendations had been identified and their impact assessed.

For the analysis, we clustered the recommendations as low (score < 40), medium (score 40–70) and high impact (score > 70).

## Results

### Delphi study

The first round (July–August 2020) involved 35 academic researchers, with a variety of backgrounds and experiences of conducting collaborative research involving various groups with characteristics of vulnerability (Table 2). Some had additional experience of being members of ethics committees and commissioning research programmes. In total, 28 of these took part in the second round (retention rate 80%), displaying a distribution of characteristics similar to those in the first round.

**Table 2:** Participants in the Delphi study

	Round 1	Round 2
Gender		
Female	26	21
Male	8	6
Other	1	1
Role		
Academic	27	22
Member of an HRA REC	3	2
Member of a University REC	4	4
Research programme manager	2	1
PhD student	1	1
Research fellow	1	1
Academic discipline		
Social care research	18	16
Health service research	17	15
Public health research	8	6
Anthropology	6	6
Sociology	6	5
Psychology	5	4
Public policy	2	2
Other^a^	5	3
Years of experience conducting research		
Less than 5	2	1
Between 5 and 10	8	7
More than 10	25	20
Years of experience conducting collaborative research		
Less than 5	6	4
Between 5 and 10	12	9
More than 10	11	9
Not applicable	6	6
Years of experience conducting collaborative research with vulnerable groups		
Less than 5	6	4
Between 5 and 10	12	10
More than 10	13	10
Not applicable	4	4
Vulnerable groups involved in the research conducted by the participants		
Individuals with mental health problems	17	13
Individuals with acquired cognitive impairment	13	11
Individuals with intellectual disabilities	12	9
Migrants and asylum seekers	7	7
Individuals experiencing homelessness	6	4
Victims/survivors of domestic violence	6	5
Children and young people	4	3
Prisoners	2	2
Other^b^	13	9
Research approaches used by the respondents		
Co-production	20	19
Co-design	18	15
Co-creation	12	10
Community-based participatory research	10	7
Participatory action research	8	6
Other^c^	6	6
Total number of times respondents have applied to health or social care research ethics committees (not university only)		
Not at all	3	3
Once	2	2
Between 2 and 5	18	13
More than 5	12	10
Number of respondents who reported applying to health or social care research ethics committees (excluding university-only committees) for approval within the last 3 years		
Yes	26	21
No	9	7

^a^Including bioethics, psychiatry, social policy, social work/family work, sport and exercise sciences

^b^Including people at the end of life, survivors of human trafficking, individuals with developmental disabilities, care leavers, ethnic minorities, families involved with child protection services, individuals with other invisible disabilities, older people, recently bereaved individuals, survivors of sexual violence, young offenders

^c^Including service user led research, Experience-based Co-Design (EBCD), research studies including some activities with a collaborative feature (e.g. collaborative workshops)

In the first round, consensus was reached on 26 statements (40% of the original set). Of these, six were under theme 1 (general research ethics principles), five under theme 7 (the research protocol), five under theme 8 (seeking consent), four under theme 5 (role and competence of researchers), two under theme 2 (involvement of participants) and one under each of themes 3 (protection of participants), 6 (the working of RECs), 9 (compliance with legislation) and 12 (benefits from research). All were about improvement options, with the stem ‘The research ethics system needs to…’.

In the second round (November–December 2020), a further seven statements reached consensus, two statements under theme 1 and one under each of themes 2, 3, 6, 7 and 11. In the second round all but one of the consensused statements related to improvement options.

By the end of the second round, 33 statements (50% of the original set) had reached consensus. 32 of these concerned how the research ethics system could be changed to make it more fit-for-purpose in the context of collaborative research with ‘vulnerable’ groups (Table 3). Among these, 14 statements reached over 90% agreement.

**Table 3:** Statement for which the Delphi panel reached consensus

Themes	Statements	Distribution of respondents by level of agreement			Round in which consensus was achieved
		**Strongly agree or agree**	**Neither agree nor disagree**	**Strongly disagree or disagree**	
Theme 1 – General research ethics principles in the context of participatory research with ‘vulnerable’ groups	- The research ethics system needs to promote open dialogue amongst research ethics committees, researchers, research participants and the public about what the fundamental principles of research ethics should be and what constitutes ethical practice in research	88.6%	8.6%	2.9%	Round 1
	- The research ethics system needs to expand current ethics review frameworks to include principles of participatory research	94.3%	5.7%	0.0%	Round 1
	- The research ethics system needs to promote a more ‘relational ethics approach’ to allow researchers and research ethics committees to work through the ethical issues they encounter, on an equal basis	85.7%	11.4%	2.9%	Round 1
	- The research ethics system needs to adopt the principle of ‘situated ethics’, acknowledging that the researcher carrying out participatory research with ‘vulnerable’ groups makes on-the-spot decisions with ethical implications	82.9%	14.3%	2.9%	Round 1
	- The research ethics system needs to overcome the disconnect between the research ethics principles and the bureaucratic procedures associated with research ethics approval for participatory research with ‘vulnerable’ groups	82.9%	5.7%	11.4%	Round 1
	- The research ethics system needs to balance potential vulnerability of participants with their empowerment when assessing participatory research with ‘vulnerable’ groups	88.6%	8.6%	2.9%	Round 1
	- The research ethics system needs to adopt a flexible model that allows for addressing ethical issues at various stages of a participatory research study	92.9%	3.6%	3.6%	Round 2
	- The research ethics system needs to envisage processes to monitor participatory research studies which are given a favourable ethical opinion	92.9%	3.6%	3.6%	Round 2
Theme 2 – Involvement of participants	- The current research ethics system allows the involvement of patients, service users and the public in the design, management, conduct and dissemination of research	82.1%	17.9%	0.0%	Round 2
	- The research ethics system needs to encourage researchers to carry out research with more diverse populations	82.9%	8.6%	8.6%	Round 1
	- The research ethics system needs to implement processes which are both appropriate and feasible to ensure that vulnerable individuals can be involved in research	94.3%	2.9%	2.9%	Round 1
Theme 3 – Protection of research participants	- The research ethics system needs to adopt a more proportionate approach in the way it protects vulnerable individuals involved in participatory research	89.3%	7.1%	3.6%	Round 2
	- The research ethics system needs to allow the researcher to use simple and proportionate processes when presenting the benefits and risks of participation to vulnerable participants	82.9%	14.3%	2.9%	Round 1
Theme 5 – Role and competence of researchers	- The research ethics system needs to acknowledge the role of the researcher in fostering the confidence that patients, service users and the public have in research	85.7%	8.6%	5.7%	Round 1
	- The research ethics system needs to support researchers to develop trusting relationships with vulnerable individuals taking part in participatory research	91.4%	0.0%	8.6%	Round 1
	- The research ethics system needs to help researchers to identify and deal, in an intellectually stimulating way, with the ethical challenges of the research	85.7%	11.4%	2.9%	Round 1
	- The research ethics system needs to support the reflexivity of researchers conducting participatory research with vulnerable groups	82.9%	11.4%	5.7%	Round 1
Theme 6 – The working of RECs	- The research ethics system needs to designate specialist research ethics committees with expertise in reviewing participatory research involving vulnerable groups	85.7%	3.6%	10.7%	Round 2
	- The research ethics system needs to periodically audit documents and decisions of specialized committees for participatory research with vulnerable groups to help to achieve consistency and high quality decision-making	80.0%	11.4%	8.6%	Round 1
Theme 7 – Research protocol	- The research ethics system needs to include explicit terminology about participatory research in the templates/forms used for research proposals/protocols and related guidelines	80.0%	14.3%	5.7%	Round 1
	- The research ethics system needs to ask researchers conducting participatory research to outline in the research proposal/protocol the intended nature of the collaborative/participatory elements of their research	91.4%	5.7%	2.9%	Round 1
	- The research ethics system needs to allow the research proposal/protocol to describe explicitly the elements of the study open to modification/development and stipulate the nature of the facilitation process through which this will occur	91.4%	5.7%	2.9%	Round 1
	- The research ethics system needs to consider approval of participatory research proposals/protocols in stages that match the unfolding of the research process	89.3%	3.6%	7.1%	Round 2
	- The research ethics system needs to allow some prudential flexibility in the requirements of the research proposal/protocol to accommodate the emergent nature of participatory research with vulnerable groups	94.3%	2.9%	2.9%	Round 1
	- The research ethics system needs to acknowledge that participatory research, by its nature, encompasses a layer of everyday ethics which cannot be comprehensively addressed in a research proposal/protocol	80.0%	11.4%	8.6%	Round 1
Theme 8 – Seeking consent	- The research ethics system needs to frame consent as an ongoing process, which is negotiated at different points throughout the research process	94.3%	2.9%	2.9%	Round 1
	- The research ethics system needs to allow some degree of personalization of consent seeking processes to ensure that they are implemented in a way that affords autonomy and dignity to potential research participants	91.4%	5.7%	2.9%	Round 1
	- The research ethics system needs to allow vulnerable participants to choose whether they would like to have someone with them when they are taking part in research, without assuming that they need or want to have someone	97.1%	0.0%	2.9%	Round 1
	- The research ethics system needs to allow more flexibility in the format of participant information sheets required for participatory research with vulnerable groups	91.4%	5.7%	2.9%	Round 1
	- The research ethics system needs to be open to considering alternatives to the signing of consent forms to accommodate participants from vulnerable groups, to afford both protection and opportunity to participate	97.1%	0.0%	2.9%	Round 1
Theme 9 – Compliance with legislation	- The research ethics system needs to adopt a flexible approach that allows researchers conducting participatory research with vulnerable groups to accommodate the needs of potential participants (e.g. to ensure transparency statements about General Data Protection Regulation - GDPR are understandable), while ensuring compliance with current legislation	97.1%	0.0%	2.9%	Round 1
Theme 11 – Accessible findings	- The research ethics system needs to establish the sharing of findings of participatory research with vulnerable groups as part of the research proposal/protocol approval process	82.1%	14.3%	3.6%	Round 2
Theme 12 – Benefits of research	- The research ethics system needs to recognize the contribution of participatory research with vulnerable groups in generating knowledge that could benefit those groups and science and/or society	88.6%	8.6%	2.9%	Round 1

At least one statement in each theme did not reach consensus. The majority of the statements which did not reach consensus (25 out of 33) referred to how the current research ethics system deals with collaborative research involving ‘vulnerable’ groups (Table 4). Among the statements that did not reach consensus, 10 had responses distributed equally across agreement, disagreement and indifference, whereas seven achieved borderline consensus (i.e. between 70 and 80%) after the second round.

**Table 4:** Statements for which the Delphi panel did not reach consensus

Themes	Statements	Distribution of respondents by level of agreement		
		**Strongly agree or agree**	**Neither agree nor disagree**	**Strongly disagree or disagree**
Theme 1 – General research ethics principles in the context of participatory research with vulnerable groups	- The current research ethics system enables and supports the undertaking of ethical participatory research in health and social care involving vulnerable groups	14.3%	25.0%	60.7%
	- The current research ethics system effectively monitors participatory research studies involving vulnerable groups which are given a favourable ethical opinion	0.0%	32.1%	67.9%
Theme 2 – Involvement of participants	- The current research ethics system allows the involvement of vulnerable individuals in the design, management, conduct and dissemination of participatory research	46.4%	25.0%	28.6%
	- The research ethics system needs to ensure that the researchers have considered engagement with key individuals or organizations that have a legitimate interest in the conduct or outcomes of the proposed participatory research	78.6%	10.7%	10.7%
Theme 3 – Protection of research participants	- The current research ethics system adequately protects the rights, safety, dignity and wellbeing of vulnerable participants involved in participatory research	46.4%	25.0%	28.6%
	- The current research ethics system weighs proportionately any anticipated benefit for the individual participant and present and future recipients of the health or social care against the foreseeable risks and inconveniences once they have been mitigated	10.7%	42.9%	46.4%
	- The research ethics system needs to assess the potential risks and benefits to communities, beyond the risk to the individual participant	78.6%	14.3%	7.1%
	- The research ethics system needs to recognize and accept the steps proposed by the researchers to ensure power-sharing when conducting participatory research with vulnerable groups	78.6%	21.4%	0.0%
Theme 4 – Privacy and confidentiality	- The current research ethics system allows the information collected in the context of participatory research to be recorded, handled and stored in an appropriate way, while adequately protecting the confidentiality of participants	67.9%	21.4%	10.7%
	- The current research ethics system allows the information collected in the context of participatory research with vulnerable groups to be recorded, handled and stored in an appropriate way, while adequately protecting the confidentiality of participants	64.3%	21.4%	14.3%
	- The research ethics system needs to allow some tolerance around confidentiality and to take a nuanced view around it	78.6%	17.9%	3.6%
Theme 5 – Role and competence of researchers	- The current research ethics system ensures that researchers undertaking participatory research with vulnerable groups are competent to pursue the proposed research or are under supervision of a competent supervisor	21.4%	25.0%	53.6%
	- The current research ethics system supports and facilitates high-quality participatory research that has the confidence of patients, service users and the public	7.1%	46.4%	46.4%
	- The research ethics system needs to trust that competent researchers, carrying out participatory research with vulnerable groups, will use an ethical listening approach in conducting their day-to-day research activities	67.9%	17.9%	14.3%
	- The research ethics system needs to be designed to empower the individual researchers to live up to their ethos	78.6%	7.1%	14.3%
Theme 6 – The working of RECs	- The current research ethics system enables relevant committees to perform an efficient and timely ethics review process when assessing participatory research involving vulnerable groups	17.9%	25.0%	57.1%
	- The current research ethics system enables relevant committees to perform a robust ethics review process when assessing participatory research involving vulnerable groups	21.4%	39.3%	39.3%
	- The current research ethics system provides proportionate ethical review when assessing participatory research involving vulnerable groups	10.7%	35.7%	53.6%
	- The research ethics system needs to introduce mechanisms for pre-review of applications of participatory research with vulnerable groups, for example, by involving the researchers and a research ethics committee (REC) or a REC member	67.9%	14.3%	17.9%
Theme 7 – Research protocol	- The current research ethics system expects the design and procedure of the research to be described in a research proposal/protocol (i.e. the document which outlines the design and procedure of the research, where applicable conforming to a standard template and/or specified content), which is fit for the purpose of participatory research with vulnerable groups	32.1%	10.7%	57.1%
Theme 8 – Seeking consent	- The current research ethics system takes meaningful and proportionate account of individual participants’ capacity to understand what research is and what participation entails when assessing proposals for participatory research with vulnerable groups	21.4%	17.9%	60.7%
	- The current research ethics system affords adequate respect to individuals from vulnerable groups who are considering whether to join/withdraw from a participatory research study	32.1%	39.3%	28.6%
	- The current research ethics system affords adequate autonomy to individuals from vulnerable groups who are considering whether to join/withdraw from a participatory research study	32.1%	21.4%	46.4%
	- The current research ethics system allows researchers to use participant information sheets fit for the purpose of enabling vulnerable individuals to choose whether to join participatory research	21.4%	25.0%	53.6%
	- The current research ethics system allows researchers to use consent forms fit for the purpose of enabling vulnerable individuals to consent to take part in participatory research	28.6%	32.1%	39.3%
Theme 9 – Compliance with legislation	- The current research ethics system expects researchers conducting participatory research with vulnerable groups to comply in a meaningful way with relevant legislation (e.g. Mental Capacity Act, 2005; Data Protection Act, 2018)	75.0%	17.9%	7.1%
Theme 10 – Integrity, quality, transparency of research	- The current research ethics system ensures that participatory research involving vulnerable groups is designed, reviewed, managed and undertaken in a way that ensures integrity	14.3%	67.9%	17.9%
	- The current research ethics system ensures that participatory research involving vulnerable groups is designed, reviewed, managed and undertaken in a way that ensures quality	21.4%	35.7%	42.9%
	- The current research ethics system ensures that participatory research involving vulnerable groups is designed, reviewed, managed and undertaken in a way that ensures transparency	32.1%	42.9%	25.0%
Theme 11 – Accessible findings	- The current research ethics system ensures that the findings of participatory research with vulnerable groups are made accessible, with adequate consent and privacy safeguards, in a timely manner	7.1%	50.0%	42.9%
	- The current research ethics system ensures that information about the findings of participatory research with vulnerable groups are available, in a suitable format and timely manner, to those who took part in it	7.1%	50.0%	42.9%
	- The research ethics system needs to suggest simple ways in which findings of participatory research with vulnerable groups can be made available to participants and other relevant stakeholders	78.6%	14.3%	7.1%
Theme 12 – Benefits of research	- The current research ethics system facilitates and promotes ethical participatory research involving vulnerable groups that is of potential benefit to those groups and to science and/or society	17.9%	53.6%	28.6%

### Final focus group

**Fig. 1:**
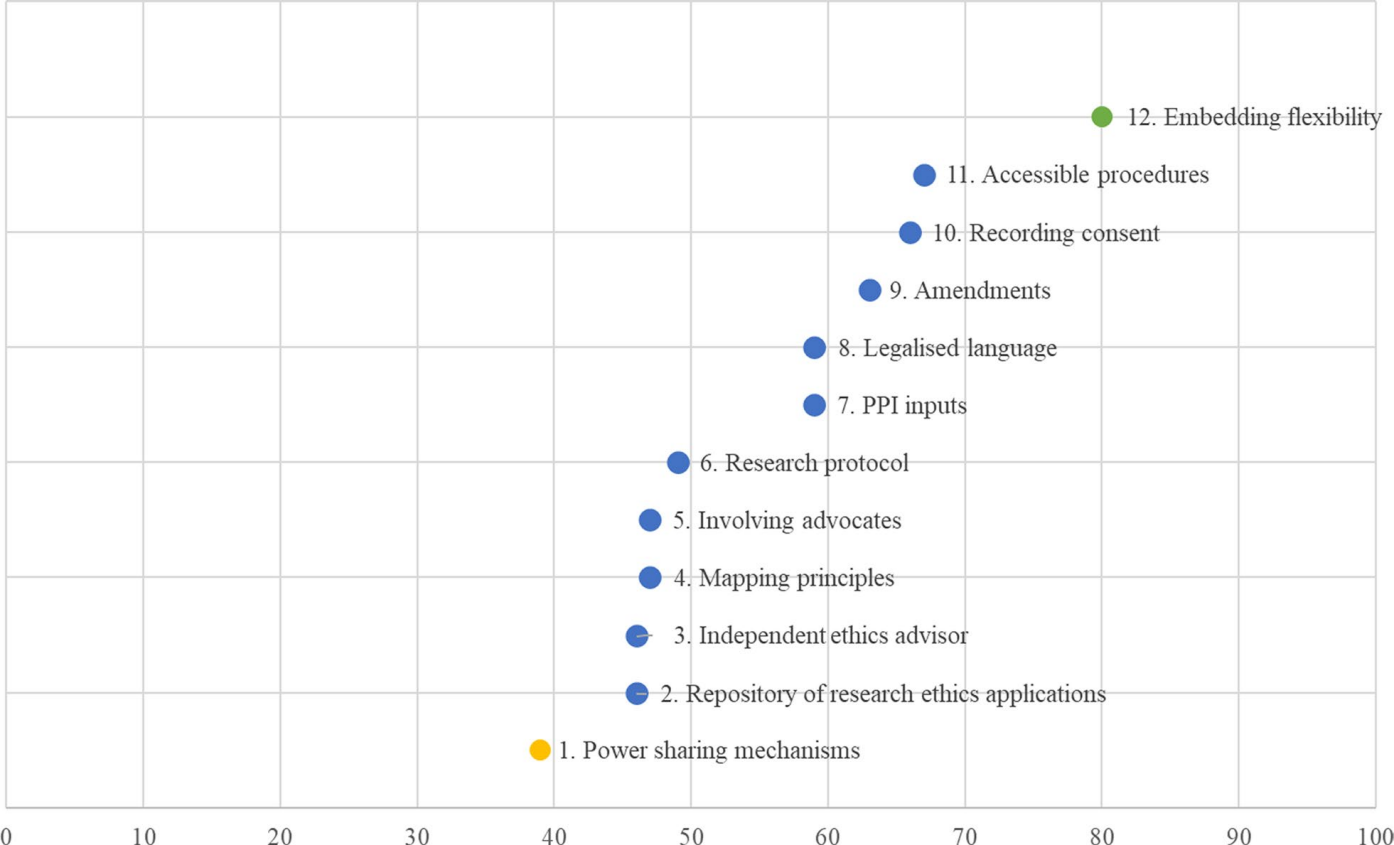
Final set of recommendations and their expected impact in ascending order

The 14 statements which gained the highest consensus in the Delphi study (i.e. above 90% of participants converged on the same level of agreement) were reviewed in the final focus group. The impact of 10 out of 12 recommendations fell in the medium range (score 40–70); one recommendation was judged to have low impact (score < 40) and one to have high impact (score > 70) (Fig. 1).

Recommendations in Fig. 1 are ranked by impact in ascending order, rather than by the order in which they were generated. Recommendation 1 (‘Power sharing mechanisms’) is to support researchers and RECs to consider how power differentials of parties to a collaborative study are managed. Collaborative research aims to address power imbalances and to privilege forms of knowledge that are not typically recognized in mainstream research. The ways in which roles and responsibilities are allocated shape how power is distributed and power differentials addressed. The recommendation is to add a section to research ethics forms to outline roles and responsibilities of the different parties and identify the power sharing mechanisms to be designed and deployed during the study. This, and other relevant sections, would be activated in the form once a ‘collaborative research project’ option was ticked by applicants.

Recommendations 2–5 received very similar scores, just below the mid-point. Recommendation 2 (‘Repository of research ethics applications’) is for the creation of a repository of collaborative research ethics applications, which could be used as reflexive tools by researchers and RECs when preparing or reviewing applications.

Recommendation 3 (‘Independent ethics advisor’) suggests the creation of the role of ethics advisor within the project but independent from the research team, who could provide advice on ethical matters and oversee the appropriate application of ethics principles as the study develops.

Recommendation 4 (‘Mapping principles’) is to map principles of collaborative research onto the principles of ethics review and onto the sections of the research ethics form. Recommendation 5 (‘Involving advocates’) aims to give participants who could be considered ‘vulnerable’ real choice about whether they would like to have someone with them when they are taking part in research, without assuming that they need, or want, to have someone.

Recommendation 6 (‘Research protocol’) is to add a section to the research ethics form where researchers describe the collaborative elements of the study (as suggested in recommendation 1) and outline how the ethical principles relate to the collaborative elements (see recommendation 4). This standardized section would help clearly articulate the key ethical issues and proposed actions to address them and enable information to be gathered consistently across studies.

Recommendation 7 (‘Patient and Public Involvement/Engagement inputs’) asks RECs to provide an explicit justification for any decision to override opinions that the research team has included in a research ethics application as a direct result of Patient and Public Involvement/Engagement activities. If the decision is grounded on an underpinning legal requirement, this should be specifically explained by the REC (e.g. by providing the extract where the specific provision is made).

Recommendation 8 (‘Legalized language’) argues that legal language should be avoided. To allow full participation in research, when reference to compliance with legislation and regulations is made, language should be accessible.

Recommendation 9 (‘Amendments’) aims to address the amendment process. The emergent design of collaborative research affects the extent to which a detailed research plan can be set out at the start of a study. Collaborative studies evolve and research plans need to be adapted accordingly. From the research ethics perspective, changes in research plans represent discrepancies from the original research plan and protocol, and therefore require a formal amendment. Recommendation 9 suggests that the research ethics committees should give full consideration to the nature of changes occurring in the course of a collaborative study and distinguish between changes that reflect how a study evolves over time and changes that represent a substantial deviation from the original research plan and its underpinning ethical principles. The system should take a proportionate approach in assessing such changes (e.g. with respect to risk to participants, if the change in the research does not pose different and additional risks to participants an amendment should not be required). The amendment process should be simplified. For example, the REC chair could take responsibility for allocating the study to a REC member who assesses whether any of the fundamental principles that were agreed at the first review are undermined. Such a way of organizing the amendment process would also contribute towards framing research ethics as a relational, ongoing and reflexive process pivoted around dialogue between research ethics committees and research teams. A streamlined, simplified and flexible amendment process could allow researchers to update the research protocol as and when changes occur and turning points are reached.

Recommendation 10 (‘Recording consent’) suggests that research ethics systems should consider alternatives to signing consent forms and make reasonable adjustments to the process to accommodate participants’ abilities and preferences, to afford them both protection and opportunity to participate.

Recommendation 11 (‘Accessible procedures’) makes a case for reviewing and revising current research ethics procedures and forms in consultation with representatives from ‘vulnerable’ groups. Co-researchers from vulnerable groups may find current research ethics systems cumbersome and inaccessible, resulting in limited opportunities for them to contribute to the application process, which then continues de facto to be led by the researchers.

Recommendation 12 (‘Embedding flexibility’) suggests reworking protocol templates to introduce flexibility in certain elements to allow research teams to account for the emergent design of the study and outline the elements which are not fully pinned down before a study starts. For example, protocols could offer an option for researchers to state the maximum burden to research participants.

## Discussion

Research ethics as a form of regulatory oversight reflects concerns about the ethical quality of research that involves human participants in an attempt to protect them from potentially unethical and even harmful research. Originally established in the biomedical field, the research ethics principles of respect for autonomy, justice, beneficence and non-maleficence have become progressively embedded in various regulatory frameworks (international, national, institutional), translated into organizational processes, formalized in procedures and embodied in professional roles. They are now also applied widely to social sciences research. ‘Ethics creep’, whereby the regulatory system has been expanding outwards, to incorporate into existing processes new research activities and institutions, as well as inwards, intensifying the regulation of activities deemed to fall within its scope [12], has been linked to a range of negative or unwanted consequences [11, 12, 46, 66–69], without any substantial empirical evidence around whether and how research ethics actually achieves what it was designed to do [67, 70, 71].

This work has identified a set of specific and practical recommendations that could contribute to a better fit between the principles and practices of collaborative research involving participants deemed vulnerable and principles underpinning research ethics frameworks and related operational processes. The recommendations were generated using a structured and robust elicitation process that allowed for consolidation of the individual views and experiences of a large and diverse group of active academic researchers with relevant experience in this field.

In taking this structured approach, our work overcomes the limitations of two different bodies of previous research and analysis. The first one comprises studies that take a case-based approach to identify issues and limitations of the current research ethics system when dealing with collaborative research (e.g. [19, 26, 72]). These result in in-depth but fragmented accounts whose transferability across settings, countries and disciplines is unclear. The second stream of research comprises articles which provide recommendations for change developed by a group of experts opportunistically convened to collectively reflect on their individual experiences of navigating the research ethics system, without following a rigorous formal process (e.g. [73]).

As part of the Delphi study, we were also able to establish the views of academic researchers in relation to how the research ethics system currently deals with collaborative research. The low level of agreement among Delphi participants on statements around the current system suggests that researchers have varying experiences with the research ethics system when seeking approval for collaborative research studies. Although our data do not explain such heterogeneity, this finding seems to suggest that how researchers experience the ethics system could be shaped cumulatively by the characteristics of the researchers themselves (e.g. their experience), of the RECs (e.g. its membership and the experience and expertise of REC members) and of the research study for approval (e.g. study design, population). Future work should further explore such heterogeneity to explain whether it constitutes warranted variation or it signals lack of consistency in the way collaborative research is reviewed by RECs.

Despite reporting contrasting experiences of the current system, participants converged on a range of topics or areas requiring improvement. Nearly all statements that reached consensus were around improvements and emerged in the first round of the Delphi process, suggesting that such options were non-controversial across wide cross-sections of the respondents, regardless of their individual experiences.

The final focus group aimed to translate the improvement options into practical, impactful recommendations that could be considered by researchers, RECs and research institutions. The 12 recommendations can be seen as comprising three clusters. Those under the first cluster suggest practical ways in which the research ethics system could ensure that the collaborative dimension of collaborative research is truly endorsed whilst also maintaining an ethical reflexivity to the conduct of the study. Recommendations for the ethics system to focus on power-sharing mechanisms, envision the role of advocates of research participants, embed Patient and Public Involvement/Engagement inputs in the design of a study and revise the language used in supporting documents (e.g. information sheets or consent forms), work towards ensuring that researchers are allowed to undertake studies which are genuinely participatory, inclusive and empowering for participants.

Recommendations under the second cluster address the overly standardized process and rigid forms required by the research ethics system. They advocate for some helpful revisions to the forms, but also a greater degree of flexibility in the process. Protocol templates currently in use do not allow articulation of how the collaborative nature of the study will impact on research activities, their intensity or their sequence. Hence, the recommendations propose adaptation of the templates to allow for description of the collaborative features of the study, alongside the research activities planned.

Recommendations in the last cluster make suggestions that could strengthen the relational and ongoing nature of ethical research practice. This could be achieved both by creating new roles (e.g. the independent ethics advisor who ideally has experience or knowledge of collaborative research) or by allocating more specific responsibilities to existing roles (e.g. when a nominated member of a REC takes responsibility for reviewing an ethics application and subsequent amendments). Both examples could support research teams and/or RECs by providing advice on specific ethics matters and overseeing the appropriate application of ethics principles as the study develops. Although it is worth acknowledging the mixed results of similar attempts (e.g. [74]), there seems to be scope for learning from previous experiences and fine-tuning them to the needs or specificities of collaborative research.

This work has some strengths and limitations. The study has been guided by three intertwined methodological choices. First, it used a theory-informed conceptual framework: its organizing categories of ‘principles’ and ‘processes’ were derived from the UK research framework and related policies, regulations and guidance, and were used systematically across the preparatory phases of the work that informed the development of the Delphi questionnaire. This ensures that the final recommendations are grounded in and relevant to the current UK policy and regulatory environment, but their applicability to different settings should be explored.

Second, the study used an exploratory mixed-methods research design, with an iteration of the sequence qualitative + quantitative, integrated by a final qualitative phase. The evidence synthesis and the initial qualitative component, reported in [75], informed the development of the Delphi questionnaire [62]. Funnelling was the organizing strategy of the study, with the results generated by using one method in one phase feeding into the next phase.

Third, our analytical strategy used an iterative process of zooming in and zooming out, from empirical data to the conceptual framework and back, to ensure the coherence of the emerging findings with the theoretical and normative footprint of the work.

The Delphi method represents a strength of the study by providing a robust process to elicit individual preferences or opinions and transform them into a group consensus [76]. Although the sample of the Delphi panel was designed to achieve variation in the level of experience (e.g. including research students, early- and middle- career and senior researchers and academics), disciplines, study populations and research approaches, the actual members of the panel were a self-selected group of those invited. The study was carried out during the coronavirus disease (COVID) pandemic, which may have further affected participant recruitment. Nevertheless, the panel comprised researchers from a range of disciplinary backgrounds and experiences of collaborative research and/or participant groups, which should ensure the relevance of the results across specific areas of expertise.

The Delphi questionnaire asked participants to rate their experiences and make suggestions for research that would sit neatly at the intersections of collaborative research and vulnerable populations. Participants may have found it difficult or artificial to disentangle their views on this specific topic from their broader research experiences. The contingent context may have also heightened participants’ sensitivities on the topic, since from the start of the pandemic research ethics processes and requirements were adjusted to reflect and respond to the unprecedented circumstances in which research was conducted. It is also worth mentioning that the working definitions of the specific possible approaches to collaborative research may have been interpreted differently by panel members, not least because terminology is far from consistent. However, given the background and experience of the Delphi panel, we feel that they shared a broad understanding of collaborative research as an umbrella term for carrying out research *with* rather than *on* people.

The Delphi study achieved a high retention rate. It also used a high threshold for consensus (80%), which helped identify agreement among wide cross-sections of the panel rather than groups of researchers with niche research interests. Although the study reached consensus on about half of the initial set of statements, across two Delphi rounds, about as many statements lacked consensus. However, the research team did not have capacity to explore and explain this aspect. 

Lastly, the study involved only researchers employed in English Universities, who provide a partial, albeit important, perspective on the UK research ethics system. The next part of this study will aim to collect the perspectives of research participants, research funders and research ethics committees to help generate a system-wide perspective of research ethics on collaborative research involving ‘vulnerable’ groups.

## Conclusions

The poor alignment between the current research ethics system, which originated in the biomedical field with clear positivist underpinnings and an orientation towards experimental research, and the aims and methods of research with different epistemological stances using qualitative or mixed-methods, has been established in the scholarly debate. Particular questions have been raised about whether and how the principles of research ethics are ultimately fulfilled when applied to collaborative research involving populations perceived to have characteristics of vulnerability.

The work reported here moves these debates forward and identifies recommendations that could improve how research ethics addresses collaborative research both from a procedural point of view, by making sure that fit-for-purpose processes are in place, and from a substantive point of view, by questioning the ethical dimensions of the choices and practices of collaborative research.

## Disclaimer

Some of the time of authors of this paper is funded by the NIHR School for Social Care Research. The views expressed are those of the author(s) and not necessarily those of the NIHR or the Department of Health and Social Care.

## Supplementary Information

Additional file 1.

Additional file 2.

## Data Availability

The data used and/or analysed during the current study are available from the corresponding author on reasonable request.
