# Supplementary material for: Fitting a square peg in a round hole? A mixed-methods study on research ethics and collaborative health and social care research involving ‘vulnerable’ groups
Source: Health Res Policy Syst. 2025 Apr 1;23:40. doi: 10.1186/s12961-025-01290-3 (PMC11963353; doi:10.1186/s12961-025-01290-3)
Supplement: Supplementary file 1 — Additional file 1. [file 12961_2025_1290_MOESM1_ESM.pdf]

Improving the research ethics for health and social care – Delphi study

Questionnaire - Round 1

Thank you for your interest in this Delphi study. With this work we aim to identify recommendations for improving the research ethics and governance system for health and social care in the UK in the context of participatory research (broadly defined) with vulnerable adults[1].

In the following pages you will find a set of statements organized in 12 sections which represent key themes on this topic. These have been generated through an iterative process, which started with an exploratory focus group with experts in the field, complemented by an analysis of policy documents underpinning the UK research ethics framework, a rapid review of the literature on the topic, and discussions with Dr Philly Hare, who supports a DEEP Dementia Enquirers' Project in which people living with dementia have developed The Ethics Gold Standards for Dementia Research.

You are invited to express your level of agreement with each statement, using a 5-point Likert scale. If you want to provide your comments or suggest further statements relevant to a specific theme, please use the space provided at the end of each section.

For each theme you can refer to some explanatory notes that provide key definitions and additional background information relevant to that theme, including the evidence base used to produce the statements (e.g. the focus group data are marked with [FG], relevant references are provided in the reference list). The explanatory notes can be accessed by clicking on the hyperlinked title of each section.

A Back Button is available to help you navigate across different sections of the questionnaire and change your responses. If you do not finish the survey in one sitting, you can retrieve your partial response by clicking on the survey link you received. You will be able to re-start from the point at which you stopped.

If you experience any problem with this form, please email Chiara De Poli (c.de-poli@lse.ac.uk).

[1] The UK Research Governance Framework conveys a consent-based conceptualisation of vulnerability, labelling adults who may have issues with understanding and decision-making as vulnerable, but does not provide an ethical framework or guiding principles relative to vulnerability. Given the absence of a formal definition of vulnerability, in this work we take the view that vulnerability is individual and situational and as such should be evaluated with respect to the context and to the characteristics of individual research participants. In doing so, we support the claim that vulnerability cannot be defined or operationalized with a labelling or sub-population approach, which perpetuate the stereotyping or stigmatising accounts of vulnerability that this work tries to challenge.

Theme 1 - General research ethics principles in the context of participatory research with vulnerable groups

Statement	Strongly agree	Agree	Neither agree nor disagree	Disagree	Strongly disagree
1. The current research ethics system enables and supports the undertaking of ethical participatory research in health and social care involving vulnerable groups.					
2. The current research ethics system effectively monitors participatory research studies involving vulnerable groups which are given a favourable ethical opinion.					
3. The research ethics system needs to promote open dialogue among Research Ethics Committees, researchers, research participants and the public about what the fundamental principles of research ethics should be and what constitutes ethical practice in research.					
4. The research ethics system needs to expand current ethics review frameworks to include principles of participatory research.					
5. The research ethics system needs to promote a more ‘relational ethics approach’ to allow researchers and Research Ethics Committees to work through the ethical issues they encounter, on an equal basis.					
6. The research ethics system needs to adopt the principle of ‘situated ethics’, acknowledging that the researcher carrying out participatory research with vulnerable groups makes on-the-spot decisions with ethical implications.					
7. The research ethics system needs to overcome the disconnect between the research ethics principles and the bureaucratic procedures associated					

Statement	Strongly agree	Agree	Neither agree nor disagree	Disagree	Strongly disagree
with research ethics approval for participatory research with vulnerable groups.					
8. The research ethics system needs to balance vulnerability of participants with their empowerment when assessing participatory research with vulnerable groups.					
9. The research ethics system needs to adopt a flexible model that allows for addressing ethical issues at various stages of a participatory research study.					
10. The research ethics system needs to envisage processes to monitor participatory research studies which are given a favourable ethical opinion.					

Please use this space to provide any comments or suggest further statements relevant to this specific theme.

Theme 2 - Involvement of research participants

Statement	Strongly agree	Agree	Neither agree nor disagree	Disagree	Strongly disagree
1. The current research ethics system allows the involvement of patients, service users and the public in the design, management, conduct and dissemination of research.					
2. The current research ethics system allows the involvement of vulnerable individuals in the design, management, conduct and dissemination of participatory research.					
3. The research ethics system needs to encourage researchers to carry out research with more diverse populations.					
4. The research ethics system needs to ensure that the researchers have engaged with any individual or organisation that has a legitimate interest in the conduct or outcomes of the proposed participatory research.					
5. The research ethics system needs to implement processes which are both appropriate and feasible to ensure that vulnerable individuals can be involved in research.					

Please use this space to provide any comments or suggest further statements relevant to this specific theme.

Theme 3 - Protection of research participants

Statement	Strongly agree	Agree	Neither agree nor disagree	Disagree	Strongly disagree
1. The current research ethics system adequately protects the rights, safety, dignity and well-being of vulnerable participants involved in participatory research.					
2. The current research ethics system weighs proportionately any anticipated benefit for the individual participant and present and future recipients of the health or social care against the foreseeable risks and inconveniences once they have been mitigated.					
3. The research ethics system needs to adopt a more proportionate approach in the way it protects vulnerable individuals involved in participatory research.					
4. The research ethics system needs to assess the potential risks and benefits to communities, beyond the risk to the individual participant.					
5. The research ethics system needs to allow the researcher to use simple and proportionate processes when presenting the benefits and risks of participation to vulnerable participants.					
6. The research ethics system needs to recognise and accept the steps proposed by the researchers to ensure power-sharing when conducting participatory research with vulnerable groups.					

Please use this space to provide any comments or suggest further statements relevant to this specific theme.

Theme 4 - Privacy and confidentiality

Statement	Strongly agree	Agree	Neither agree nor disagree	Disagree	Strongly disagree
1. The current research ethics system allows the information collected in the context of participatory research to be recorded, handled and stored in an appropriate way, while adequately protecting the confidentiality of participants.					
2. The current research ethics system allows the information collected in the context of participatory research with vulnerable groups to be recorded, handled and stored in an appropriate way, while adequately protecting the confidentiality of participants					
3. The research ethics system needs to allow some tolerance around confidentiality and to take a nuanced view around it.					

Please use this space to provide any comments or suggest further statements relevant to this specific theme.

Theme 5 - Role and competence of researchers

Statement	Strongly agree	Agree	Neither agree nor disagree	Disagree	Strongly disagree
1. The current research ethics system ensures that researchers undertaking participatory research with vulnerable groups are competent to pursue the proposed research or are under supervision of a competent supervisor.					
2. The research ethics system supports and facilitates high-quality participatory research that has the confidence of patients, service users and the public.					
3. The research ethics system needs to acknowledge the role of the researcher in fostering the confidence of patients, service users and the public in research.					
4. The research ethics system needs to support researchers to develop trusting relationships with vulnerable individuals taking part in participatory research.					
5. The research ethics system needs to trust that competent researchers, carrying out participatory research with vulnerable groups, will use an ethical listening approach in conducting their day-to-day research activities.					
6. The research ethics system needs to be designed to empower the individual researchers to live up to their ethos.					
7. The research ethics system needs to help the researchers to identify and deal, in an intellectually stimulating way, with the ethical challenges of the research.					

Statement	Strongly agree	Agree	Neither agree nor disagree	Disagree	Strongly disagree
8. The research ethics system needs to support the reflexivity of researchers conducting participatory research with vulnerable groups.					

Please use this space to provide any comments or suggest further statements relevant to this specific theme.

Theme 6 - The working of RECs

Statement	Strongly agree	Agree	Neither agree nor disagree	Disagree	Strongly disagree
1. The current research ethics system enables relevant committees to perform an efficient and timely ethics review process when assessing participatory research involving vulnerable groups.					
2. The current research ethics system enables relevant committees to perform a robust ethics review process when assessing participatory research involving vulnerable groups.					
3. The current research ethics system provides proportionate ethical review when assessing participatory research involving vulnerable groups.					
4. The research ethics system needs to designate specialist Research Ethics Committees with expertise in reviewing participatory research involving vulnerable groups.					
5. The research ethics system needs to introduce mechanisms for pre-review of applications of participatory research with vulnerable groups for example by involving the researchers and a Research Ethics Committee (REC) or a REC member.					
6. The research ethics system needs to periodically audit documents and decisions of specialised committees for participatory research with vulnerable groups, to help to achieve consistency and high quality decision-making.					

Please use this space to provide any comments or suggest further statements relevant to this specific theme.

Theme 7 - The research protocol

Statement	Strongly agree	Agree	Neither agree nor disagree	Disagree	Strongly disagree
1. The current research ethics system expects the design and procedure of the research to be described in a research proposal/protocol (i.e. the document which outlines the design and procedure of the research, where applicable conforming to a standard template and/or specified content) which is fit for the purpose of participatory research with vulnerable groups.					
2. The research ethics system needs to include explicitly terminology of participatory research in the templates/forms used for the research proposal/protocol and relative guidelines.					
3. The research ethics system needs to ask researchers conducting participatory research to outline in the research proposal/protocol the intended nature of the collaborative/participatory elements of their research.					
4. The research ethics system needs to allow research proposal/protocol to describe explicitly the elements of the study open to modification and stipulate the nature of the facilitation process through which this will occur.					
5. The research ethics system needs to consider approval of participatory research proposals/protocols in stages that match the unfolding of the research process.					
6. The current research ethics system needs to allow some prudential flexibility in the requirements of the research proposal/protocol. to accommodate the emergent nature of					

Statement	Strongly agree	Agree	Neither agree nor disagree	Disagree	Strongly disagree
participatory research with vulnerable groups.					
7. The research ethics system needs to acknowledge that participatory research, by its nature, encompasses a layer of everyday ethics which cannot be comprehensively addressed in a research proposal/protocol.					

Please use this space to provide any comments or suggest further statements relevant to this specific theme.

Theme 8 - Seeking consent

**Statement**	**Strongly agree**	**Agree**	**Neither agree nor disagree**	**Disagree**	**Strongly disagree**
1. The current research ethics system takes meaningful and proportionate account of individual participants' capacity to understand what research is and what participation entails when assessing proposals for participatory research with vulnerable groups					
2. The current research ethics system affords adequate <u>respect</u> to individuals from vulnerable groups who are considering whether to join/withdraw from a participatory research study.					
3. The current research ethics system affords adequate <u>autonomy</u> to individuals from vulnerable groups who are considering whether to join/withdraw from a participatory research study.					
4. The current research ethics system allows researchers to use Participant Information Sheets fit for the purpose of enabling vulnerable individuals to choose whether to join participatory research.					
5. The current research ethics system allows researchers to use Consent Forms fit for the purpose of enabling vulnerable individuals to consent to take part in participatory research.					
6. The research ethics system needs to frame consent as an ongoing process, which is negotiated at different points throughout the research process.					
7. The research ethics system needs to allow some degree of personalization of the consent seeking processes to ensure that they are implemented in a					

Statement	Strongly agree	Agree	Neither agree nor disagree	Disagree	Strongly disagree
way that affords autonomy and dignity to research participants.					
8. The research ethics system needs to allow vulnerable participants to choose whether they would like to have someone with them when they are taking part in research, without assuming that they need or want to have someone.					
9. The research ethics system needs to allow more flexibility in the format of Participant Information Sheets required for participatory research with vulnerable groups.					
10. The research ethics system needs to be open to considering alternatives to the signing of consent forms to accommodate participants from vulnerable groups, to afford both protection and opportunity to participate.					

Please use this space to provide any comments or suggest further statements relevant to this specific theme.

Theme 9 - Compliance with legislation

Statement	Strongly agree	Agree	Neither agree nor disagree	Disagree	Strongly disagree
1. The current research ethics system expects researchers conducting participatory research with vulnerable groups to comply in a meaningful way with relevant legislation (e.g. Mental Capacity Act, 2005; Data Protection Act, 2018)					
2. The research ethics system needs to adopt a flexible approach that allows researchers conducting participatory research with vulnerable groups to accommodate the needs of potential participants (e.g. to ensure transparency statements on GDPR are understandable), while ensuring compliance with current legislation					

Please use this space to provide any comments or suggest further statements relevant to this specific theme.

Theme 10 - Integrity, quality, transparency of research

Statement	Strongly agree	Agree	Neither agree nor disagree	Disagree	Strongly disagree
1. The current research ethics system ensures that participatory research involving vulnerable groups is designed, reviewed, managed and undertaken in a way that ensures <u>integrity.</u>					
2. The current research ethics system ensures that participatory research involving vulnerable groups is designed, reviewed, managed and undertaken in a way that ensures <u>quality.</u>					
3. The current research ethics system ensures that participatory research involving vulnerable groups is designed, reviewed, managed and undertaken in a way that ensures <u>transparency.</u>					

Please use this space to provide any comments or suggest further statements relevant to this specific theme.

Theme 11 - Accessible findings

Statement	Strongly agree	Agree	Neither agree nor disagree	Disagree	Strongly disagree
1. The current research ethics system ensures that the findings of participatory research with vulnerable groups are made accessible, with adequate consent and privacy safeguards, in a timely manner.					
2. The current research ethics system ensures that information about the findings of participatory research with vulnerable groups are available, in a suitable format and timely manner, to those who took part in it.					
3. The research ethics system needs to suggest simple ways in which findings of participatory research with vulnerable groups can be made available to participants and other relevant stakeholders.					
4. The research ethics system needs to establish sharing of findings of participatory research with vulnerable groups as part of the research proposal/protocol approval.					

Please use this space to provide any comments or suggest further statements relevant to this specific theme.

Theme 12 - Benefits from research

Statement	Strongly agree	Agree	Neither agree nor disagree	Disagree	Strongly disagree
1. The current research ethics system facilitates and promotes ethical participatory research involving vulnerable groups that is of potential benefit to those groups and to science and/or society.					
2. The research ethics system needs to recognize the contribution of participatory research with vulnerable groups in generating knowledge that could benefit these groups and science and/or society.					

Please use this space to provide any comments or suggest further statements relevant to this specific theme.

Improving the research ethics system for health and social care - Delphi study

Questionnaire - Round 1
Explanatory notes

Definitions

- Vulnerable group. In this study we take the view that vulnerability is individual and situational and, as such, should be evaluated with respect to the context and to the characteristics of individual research participants. Following on this, we assume that individuals may be rendered vulnerable by disease or disability or by personal, societal or environmental conditions which may affect their decision making or may make them at higher risk to harm or to undue influence, coercion or exploitation.[1,2] In the following statements we use the expression vulnerable groups to refer to those participants whose individual circumstances have been considered and identified for making them vulnerable.
- Participatory research. We refer to participatory research in a broad sense to include action research, community-based participatory research and collaborative research approaches, such as co-production, co-design, co-creation, in which participants are involved in an active way, beyond simply providing data.[3,4]
- Research ethics system. We refer to research ethics system as the set of organizations (e.g. national-level Research Ethics Committees (RECs) administered by the Health Research Authority, local clinical governance or research and development (R&D) offices, departmental, school, faculty and university-wide RECs) that deal with research ethics and governance in the field of health and social care research, their arrangements and practices.

Theme 1 - General research ethics principles in the context of participatory research with vulnerable groups

The Health Research Authority (HRA) is committed to enabling and supporting ethical research in the NHS[5] and to reviewing the favourable ethical opinion given to any research study.[6]

This set of statements explores how the research ethics system currently approaches participatory research involving vulnerable groups and suggests possible directions along which the system could be improved, such as

- Promoting dialogue among researchers, RECs and research participants about ethics principles and ethical practice in participatory research[1,7–15, FG]
- Expanding the current ethics review frameworks to include principles of participatory research[13]
- Adopting a relational approach to reviewing proposals for participatory research, which could encourage a constructive discussion between researchers and RECs around their respective needs and expectations while fostering transparency and reciprocity in the review process[8,11,16]
- Adopting the principle of "situated ethics", according to which ethical considerations and decisions are conditional according to the situation and happen on the spot, and cannot be anticipated prospectively in full[7,17, FG]
- Balancing the tension between vulnerability of research participants to exploitation and their meaningful empowerment[18,19]
- Adopting a flexible model that allows ethical issues to be addressed at various stages, as they arise during each phase of a participatory research study, rather than through a one-time review. This could harness the emergent nature of participatory research, which often makes it impossible to articulate with accuracy the details of a study at the outset and/or to anticipate the variety of ethical issues that researchers will face when in the field[11,15,20]
- Envisaging processes aiming at monitoring in a meaningful way the participatory studies which received a favourable opinion[21]

Theme 2 – Involvement of participants

The set of statements around the involvement of participants in research is grounded in the UK Policy Framework for Health and Social Care, Principle 4, Patient, service user and public involvement: "Patients, service users and the public are involved in the design, management, conduct and dissemination of research, unless otherwise justified".[22]

Some of these statements question whether and to what extent the research ethics system applies this principle when reviewing participatory research involving vulnerable groups,[23,FG] and more broadly, the extent to which the current research ethics system supports researchers in carrying out participatory research which is truly inclusive and diverse.[24, FG]

Theme 3 – Protection of research participants

The set of statements builds on the HRA's commitment to protect the rights, safety, dignity and well-being of research participants[5] and is grounded in the UK Policy Framework for Health and Social Care, Principle 1, Safety: "The safety and well-being of the individual prevail over the interests of science and society" and Principle 8, Risks and benefits: "Before the research project is started, any anticipated benefit for the individual participant and other present and future recipients of the health or social care in question is weighed against the foreseeable risks and inconveniences once they have been mitigated".[22]

The statements explore how the research ethics system currently protects vulnerable individuals taking part in participatory research and suggests possible directions along which the system could be improved, such as:

- Adopting a more proportionate approach to the way the system protects vulnerable individuals willing to take part in research[1,11,13,21,25,26, FG]
- Assessing benefits and risks associated with the research with respect to the communities to which research participants belong[13,15]
- Simplifying the processes in place to present the benefits and risks of participation to potential participants[25]
- Adopting mechanisms that could ensure power-sharing when conducting participatory research with vulnerable groups[1,14]

Theme 4 – Privacy and confidentiality

The set of statements around privacy and confidentiality is grounded in the UK Policy Framework for Health and Social Care, Principle 14, Respect for Privacy: "All information collected for or as part of the research project is recorded, handled and stored appropriately and in such a way and for such time that it can be accurately reported, interpreted and verified, while the confidentiality of individual research participants remains appropriately protected. Data and tissue collections are managed in a transparent way that demonstrates commitment to their appropriate use for research and appropriate protection of privacy".[5]

The statements explore how the research ethics system currently protects the confidentiality of individuals from vulnerable groups taking part in participatory research. It also explores whether the research ethics system needs to allow some tolerance around confidentiality and to take a nuanced view around it. For example, it suggests whether it should be possible to differentiate anonymity and confidentiality[11,17] and to find ways to balance participants' desire to be identified, so that their voices are heard, with concerns for their adequate protection.[27,28]

Theme 5 – Role and competence of researchers

The UK Policy Framework aims to facilitate high-quality research in health and social care so that it has the confidence of patients, service users and the public.[22] Its Principle 2, Competence, highlights the role of the researchers in contributing to this overarching aim: "All the people involved in managing and conducting a research project are qualified by education, training and experience, or otherwise competent under the supervision of a suitably qualified person, to perform their tasks".[22]

This set of statements explores whether and in what directions the research ethics system could enhance the role of the researchers carrying out participatory research with vulnerable groups, such as:

- Acknowledging the role of the researchers in building patients', service users' and the public's confidence in research [FG]
- Setting up processes geared towards producing trust between the researchers, the research participants and the research enterprise[25,FG]
- Trusting that in their day-to-day research activities, competent researchers can practice 'ethical listening', i.e. a blend of flexibility, structure, sensitivity, and pragmatism, conditional on the situation[25,29,FG]
- Acknowledging that individual researchers aspire to live up to their ethos and ethical principles when in the field[25,FG]
- Supporting the researchers to identify and deal with the ethical challenges that participatory research poses, in an intellectually stimulating way [FG]
- Promoting the practice of reflexivity as a key element of participatory research involving vulnerable groups, for example by keeping an ethics log or identifying opportunities for sharing and discussing ethics issues as the researchers face them[29,FG]

Theme 6 – The working of RECs

The Health Research Authority (HRA) has a duty to provide an efficient, robust, proportionate and responsive ethical review of research through Research Ethics Committees (RECs).[5]

This set of statements explores how RECs currently review participatory research involving vulnerable groups and suggests possible directions along which the system could be improved, such as

- designating specialist RECs with expertise in reviewing participatory research involving vulnerable groups[8,11,13,21, FG]
- developing mechanisms for pre-review (e.g. between researchers and a REC, or a REC member) that could serve as a guidance opportunity for researchers before

finalizing their research ethics application and to assist RECs to have a better understanding of the research ethics application they will review[12,16,30–32]

- implementing auditing mechanisms to review the outcomes of the research ethics applications reviewed by specialised RECs which could help to achieve consistency and high quality decision-making[21]

Theme 7 – Research protocol

This set of statements is grounded in the UK Policy Framework for Health and Social Care, Principle 6, Protocol, which states that "The design and procedure of the research are clearly described and justified in a research proposal or protocol, where applicable conforming to a standard template and/or specified contents".[5]

These statements explore whether the research proposals/protocols currently requested by the research ethics system allow researchers to outline adequately and meaningfully the activities planned for participatory studies. It also suggests some options for reframing the purpose and meaning of such proposals/protocols and for redesigning their content, such as:

- Explicitly adopting terminology of participatory research in the templates/forms used for the research proposal/protocol and relative guidelines[8]
- Asking researchers to outline the intended nature of the collaborative/participatory elements of the research (e.g. the roles and expectations of the various partners with regard to research process, ownership of data, and dissemination of results)[8,14,18]
- Asking researchers to describe explicitly what elements of the study are open to modification and articulate the nature of the facilitation process through which this will occur[14]
- Considering approval of proposals for participatory research in stages that match the unfolding of the research process[11,15]
- Allowing some flexibility in the requirements of protocols/research proposals to accommodate the emergent nature of participatory research, for example by allowing articulating and requesting permission for deviations from standard procedures and for waivers of normal requirements[8] or by taking a more proportionate approach in reviewing amendments,[FG] for example by assessing with some flexibility any variances from the research protocol which do not constitute a material change in risk to participants and their communities[16]
- Acknowledging that participatory research encompasses some layers of everyday ethics which cannot be comprehensively addressed in a research proposal/protocol[10,15]

Theme 8 - Seeking consent

The set of statements around consent seeking is grounded in the UK Policy Framework for Health and Social Care, Principle 12, Choice: "Research participants (either directly, or indirectly through the involvement of data or tissue that could identify them) are afforded respect and autonomy, taking account of their capacity to understand. Where there is a difference between the research and the standard practice that they might otherwise experience, research participants are given information to understand the distinction and make a choice, unless a research ethics committee agrees otherwise. Where participants' explicit consent is sought, it is voluntary and informed. Where consent is refused or withdrawn, this is done without reprisal".[22]

Some of these statements explore the key features of the process of taking consent from research participants, as currently defined by the research ethics system. They also include some options for reframing how consent is sought by researchers and given by research participants, such as:

- Framing consent as an ongoing process, which is negotiated and re-negotiated at different points throughout the research process [7,17,25,FG]
- Allowing some degree of personalization ('tolerances')[17] of the consent seeking processes to ensure that they are implemented in a way that affords real agency and dignity to study participants and that avoids paternalism towards them[19]
- Allowing real choice for research participants from vulnerable groups about involvement of proxies or consultees[25,FG]
- Using Participant Information Sheets fit for the purpose (e.g. in terms of length, complexity, language) of enabling individuals from vulnerable groups to choose whether to join participatory research and to consent to it[1,10,25,FG]
- Remaining open to waiving or considering alternatives (e.g. digital recording) to the signing of consent forms to accommodate participants from vulnerable groups, when an argument has been made by the researchers that signing conventional forms would compromise the safety of participants and create significant barriers to participation[16,32]

Theme 9 – Compliance with legislation

The set of statements explores how the current research ethics system expects researchers to comply with legislation, as per the UK Policy Framework for Health and Social Care, Principle 7 Legality: "The researchers and sponsor familiarise themselves with relevant legislation and guidance in respect of managing and conducting the research".[5]

Theme 10 - Integrity, Quality and Transparency of research

This set of statements is grounded in the UK Policy Framework for Health and Social Care, Principle 5, Integrity, Quality and Transparency: "Research is designed, reviewed, managed and undertaken in a way that ensures integrity, quality and transparency".[5]

Theme 11 - Accessible findings

This set of statements is grounded in the UK Policy Framework for Health and Social Care, Principle 11, Accessible Findings: "Other than research for educational purposes and early phase trials, the findings, whether positive or negative, are made accessible, with adequate consent and privacy safeguards, in a timely manner after they have finished, in compliance with any applicable regulatory standards, i.e. legal requirements or expectations of regulators. In addition, where appropriate, information about the findings of the research is available, in a suitable format and timely manner, to those who took part in it, unless otherwise justified".[5]

It explores how the research ethics system could support the dissemination of findings of participatory research with vulnerable groups,[15] for example by considering in what format they could be presented (e.g. the form of research bulletins, plain language summaries, or visual representations) and to what audience(s).[8]

Theme 12 - Benefits of research

This set of statements reflects the HRA Research Ethics service's mission to facilitate and promote ethical research that is of potential benefit to participants, science and society[5] and explores whether the research ethics system needs to acknowledge and value the contribution that participatory research, alongside other research paradigms and approaches, could make.[17]

References

FG Reference to the views expressed by the focus group participants

1 Opsal T, Wolgemuth J, Cross J, *et al.* 'There Are No Known Benefits . . .': Considering the Risk/Benefit Ratio of Qualitative Research. *Qual Health Res* 2016; **26**: 1137–50.

2 Bracken-Roche D, Bell E, Macdonald ME, Racine E. The concept of 'vulnerability' in research ethics: An in-depth analysis of policies and guidelines. *Heal Res Policy Syst* 2017; **15**: 1–18.

3 Bradbury-Jones C, Isham L, Taylor J. The complexities and contradictions in participatory research with vulnerable children and young people: A qualitative systematic review. *Soc Sci Med* 2018; **215**: 80–91.

4 Øye C, Sørensen NØ, Dahl H, Glasdam S. Tight Ties in Collaborative Health Research Puts Research Ethics on Trial? A Discussion on Autonomy, Confidentiality, and Integrity in Qualitative Research. *Qual Health Res* 2019; **29**: 1227–35.

5 The Health Research Authority. Research Ethics Service. 2019. https://www.hra.nhs.uk/about-us/committees-and-services/res-and-recs/research-ethics-service/.

6 The Health Research Authority. Standard Operating Procedures for Research Ethics Committees. The Health Research Authority, 2019.

7 Øye C, Sørensen NØ, Glasdam S. Qualitative research ethics on the spot: Not only on the desktop. *Nurs Ethics* 2016; **23**: 455–64.

8 Guta A, Wilson MG, Flicker S, *et al.* Are we asking the right questions? A review of Canadian REB practices in relation to community-based participatory research. *J Empir Res Hum Res Ethics* 2010; **5**: 35–46.

9 Shore N, Drew E, Brazauskas R, Seifer SD. Relationships between community-based processes for research ethics review and institution-based irbs: A national study. *J Empir Res Hum Res Ethics* 2011; **6**: 13–21.

10 Noorani T, Charlesworth A, Kite A, McDermont M. Participatory Research and the Medicalization of Research Ethics Processes. *Soc Leg Stud* 2017; **26**: 378–400.

11 McCormack D, Carr T, McCloskey R, Keeping-Burke L, Furlong KE, Doucet S. Getting through ethics: The fit between research ethics board assessments and qualitative research. *J Empir Res Hum Res Ethics* 2012; **7**: 30–6.

12 Wolf LE. The research ethics committee is not the enemy: oversight of community-based participatory research. *J Empir Res Hum Res Ethics* 2010; **5**: 77–86.

13 Tamariz L, Medina H, Taylor J, Carrasquillo O, Kobetz E, Palacio A. Are Research Ethics Committees Prepared for Community-Based Participatory Research? *J Empir Res Hum Res Ethics* 2015; **10**: 488–95.

14 Goodyear-Smith F, Jackson C, Greenhalgh T. Co-design and implementation research: challenges and solutions for ethics committees. *BMC Med Ethics* 2015; **16**: 78.

15 Cross JE, Pickering K, Hickey M. Community-Based Participatory Research, Ethics, and Institutional Review Boards: Untying a Gordian Knot. *Crit Sociol* 2015; **41**: 1007–26.

16 Guta A, Nixon S, Gahagan J, Fielden S. ‘Walking along beside the researcher’: How Canadian REBs/ IRBs are responding to the needs of community-based participatory research. *J Empir Res Hum Res Ethics* 2012; **7**: 17–27.

17 Doyle E, Buckley P. Embracing qualitative research: a visual model for nuanced research ethics oversight. *Qual Res* 2017; **17**: 95–117.

18 Gustafson DL, Brunger F. Ethics, ‘Vulnerability,’ and Feminist Participatory Action Research With a Disability Community. *Qual Health Res* 2014; **24**: 997–1005.

19 Lange MM, Rogers W, Dodds S. Vulnerability in research ethics: A way forward. *Bioethics* 2013; **27**: 333–40.

20 Whicher D, Kass N, Saghai Y, Faden R, Tunis S, Pronovost P. The views of quality improvement professionals and comparative effectiveness researchers on ethics, IRBs, and oversight. *J Empir Res Hum Res Ethics* 2015; **10**: 132–44.

21 Fiscella K, Tobin JN, Carroll JK, He H, Ogedegbe G. Ethical oversight in quality improvement and quality improvement research: New approaches to promote a learning health care system Ethics in Biomedical Research. *BMC Med Ethics* 2015; **16**: 63.

22 The Health Research Authority. UK Policy Framework for Health and Social Care Research. *Heal Res Auth* 2017; : 1–40.

23 Lavery J V. Wicked problems', community engagement and the need for an implementation science for research ethics. *J Med Ethics* 2018; **44**: 163–4.

24 Noorani T, Charlesworth A, Kite A, McDermont M. Participatory Research and the Medicalization of Research Ethics Processes. *Soc Leg Stud* 2017; **26**: 378–400.

25 DEEP. The DEEP-Ethics Gold Standards for Dementia Research. DEEP, 2020.

26 Ross LF, Loup A, Nelson RM, *et al.* Human subjects protections in community-engaged research: A research ethics framework. *J Empir Res Hum Res Ethics* 2010; **5**: 5–17.

27 Petrova M, Barclay S. Response to Correspondence from Kolstoe and colleagues concerning our paper entitled, Research approvals iceberg: How a 'low-key' study in England needed 89 professionals to approve it and how we can do better. *BMC Med Ethics* 2019; **20**: 101.

28 Yanar ZM, Fazli M, Rahman J, Farthing R. Research Ethics Committees and Participatory Action Research With Young People. *J Empir Res Hum Res Ethics* 2016; **11**: 122–8.

29 Townsend A, Cox SM, Li LC. Qualitative Research Ethics: Enhancing Evidence-Based Practice in Physical Therapy. *Phys Ther* 2010; **90**: 615–28.

30 Whicher D, Kass N, Saghai Y, Faden R, Tunis S, Pronovost P. The views of quality improvement professionals and comparative effectiveness researchers on ethics, IRBs, and oversight. *J Empir Res Hum Res Ethics* 2015; **10**: 132–44.

31 Dixon-Woods M, Foy C, Hayden C, Al-Shahi Salman R, Tebbutt S, Schroter S. Can an ethics officer role reduce delays in research ethics approval? A mixed-method evaluation of an improvement project. *BMJ Open* 2016; **6**: e011973.

32 Burns D, Hyde P, Killett A, Poland F, Gray R. Participatory Organizational Research: Examining Voice in the Co-production of Knowledge. *Br J Manag* 2014; **25**: 133–44.
